# Shifting Global Invasive Potential of European Plants with Climate Change

**DOI:** 10.1371/journal.pone.0002441

**Published:** 2008-06-18

**Authors:** A. Townsend Peterson, Aimee Stewart, Kamal I. Mohamed, Miguel B. Araújo

**Affiliations:** 1 Natural History Museum and Biodiversity Research Center, The University of Kansas, Lawrence, Kansas, United States of America; 2 Department of Biological Sciences, State University of New York at Oswego, Oswego, New York, United States of America; 3 Biodiversity and Evolutionary Biology Department, National Museum of Natural Sciences, CSIC, Madrid, Spain; Centre National de la Recherche Scientifique, France

## Abstract

Global climate change and invasions by nonnative species rank among the top concerns for agents of biological loss in coming decades. Although each of these themes has seen considerable attention in the modeling and forecasting communities, their joint effects remain little explored and poorly understood. We developed ecological niche models for 1804 species from the European flora, which we projected globally to identify areas of potential distribution, both at present and across 4 scenarios of future (2055) climates. As expected from previous studies, projections based on the CGCM1 climate model were more extreme than those based on the HadCM3 model, and projections based on the a2 emissions scenario were more extreme than those based on the b2 emissions scenario. However, less expected were the highly nonlinear and contrasting projected changes in distributional areas among continents: increases in distributional potential in Europe often corresponded with decreases on other continents, and species seeing expanding potential on one continent often saw contracting potential on others. In conclusion, global climate change will have complex effects on invasive potential of plant species. The shifts and changes identified in this study suggest strongly that biological communities will see dramatic reorganizations in coming decades owing to shifting invasive potential by nonnative species.

## Introduction

Considerable recent concern has focused on the potential negative effects of global climate change and species invasions on native biodiversity [1], [2]. Although each of these themes has seen extensive monitoring and predictive forecasting [3], [4], [5], [6], [7], [8], [9], their joint effects remain largely unexplored [10]. Although single species’ likely responses have been analyzed [11], we here provide a first exploration of climate change effects on global trends in invasive potential, based on a large sample of European plant species.

Ecological niche modeling (ENM) provides a predictive framework for anticipating spatial consequences of global change phenomena for biodiversity [12], [13]. Regarding climate change, extensive methodological testing has produced not just consistent and robust projections across future climate projections [13], [14], [15], [16], [17], [18], [19], [20], [21], but also a growing understanding of the sensitivity, assumptions, and limitations of the approach [13], [22], [23], [24]. Similarly, application of ENM to forecasting potential geographic distributions of invading species has seen extensive testing [25], [26], [27], [28], [29], [30], [31], [32], [33]. Although each topic has attracted attention individually, their combined effects have seen little or no attention [11], [34].

This study provides a first survey of likely changes in invasive potential under changing climates of a significant sample of biodiversity—in this case, we assess likely changes in the global invasive potential of >1800 species of European plants. This data set, which has been explored regarding biodiversity patterns *within* Europe [20], [35], here provides the basis for development of ENMs that can be used to learn about trends in global geographic potential of species under present and future climate regimes. This study builds on the foundation of ENM applications for assessment of global invasive potential [28], [33], but extends it significantly in assessing interactions between invasive potential and current dramatic changes in climate.

## Results

### Validation of Model Results

Of the European plant species included in this study, many are already known to be present as introduced and possibly invasive species on other continents. In spite of difficulties of making taxonomic equivalencies, we identified at least 65 species from the pool under consideration that are considered invasive in the United States (see Text S1); many more are known as alien species in other regions and continents. A first challenge for the ENMs developed in this study is thus that of predicting *present-day* distributions of European plant species invasive on other continents. That is, if the models hold significant predictive ability on other continents with distinct biotic communities, then independent occurrence information should be more coincident with projections than expected by chance.

**Figure 1:**
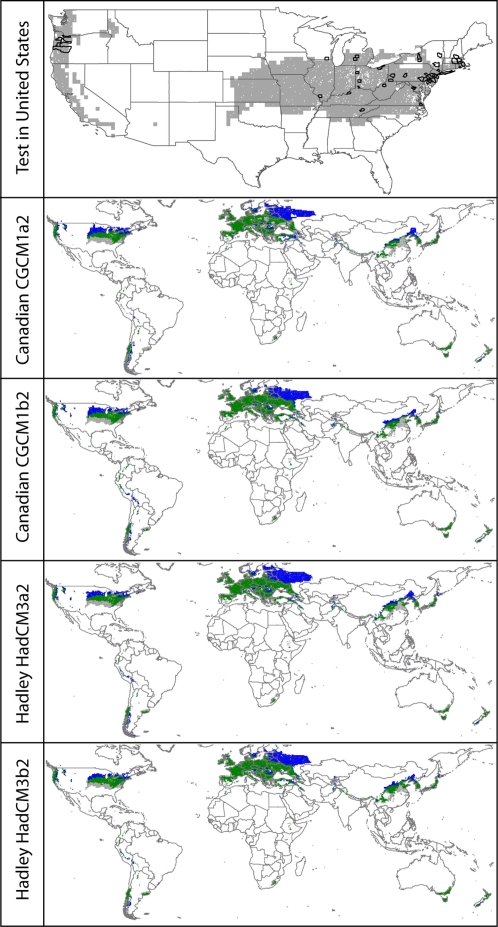
Example of projections of Ranunculus ficaria. Top panel: projection of European niche model to North America (gray shading), with United States counties from which the species is known overlaid (black outlines), illustrating the excellent coincidence between projection and independent test occurrence data. Succeeding panels show geographic trends at a global scale that are expected with future climate change under 2 scenarios each from 2 general circulation models (GCMs): gray  =  current distributional area projected to be lost with global climate change, green  =  current distributional area projected to be retained with global climate change, blue  =  areas projected to become suitable for the species with global climate change.

Hence, based on European occurrences, can we project a niche model to another continent, and anticipate the species’ distributional potential there? An example analysis, illustrated in Figure 1, is that of projections of the North American potential distribution of *Ranunculus ficaria*. Of the 3111 counties in the United States considered, 1520 were predicted as within the potential distribution of the species; of the 52 counties where this species is known to occur in the United States, 48 were among those predicted present. This level of coincidence of model projections of the species’ North American distributional potential based on European occurrences is significantly higher than expected by chance (*P* = 1.8×10^−12^). Of the 10 species tested in this way, 9 projections were statististically significant (Table 1).

**Table 1:** Summary of results of tests of intercontinental predictive ability for 10 exemplar species.

Species	Predicted present	Known present	Coincidence	*P*
*Lychnis flos-cuculi* L.	1580	83	82	<10^−10^
*Ranunculus sardous* Crantz	1615	303	166	0.15
*Brassica tournefortii* Gouan (Native to Africa)	1855	14	13	0.00072
*Carpobrotus edulis* (L.) L. Bolus (Native to southern Africa)	1668	20	18	7.1×10^−5^
*Cardamine impatiens* L.	1861	29	29	3.4×10^−7^
*Coronopus squamatus* (Forssk.) Aschers.	1314	14	9	0.027
*Silene conoidea* L.	1666	22	16	0.020
*Clematis vitalba* L.	1868	25	22	0.00044
*Clematis orientalis* L. (Native to China)	685	15	13	7.5×10^−9^
*Ranunculus ficaria* L.	1520	52	48	1.8×10^−12^

Presented are the number of counties in which the species was predicted potentially present (“Predicted present;” out of 3111), the number of counties of known occurrence (“Known present”) [54], the coincidence between the latter two sets of counties (“Coincidence”), and the probability value associated with this coincidence.

### Global Forecasts of Changing Invasive Potential

**Figure 2:**
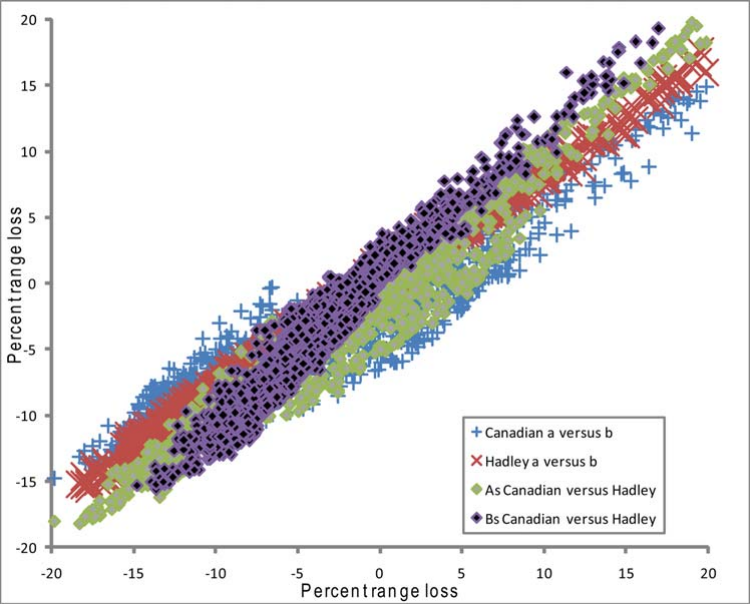
Relationship between predicted change in potential distributional area in Europe based on two atmospheric greenhouse gas scenarios (A2, which is relatively extreme in its projections for future climates, and B2, which is more conservative), and based on two general circulation models developed by two climate modeling centers (Hadley Centre, Canadian Climate Center).

Predicted change in potential distributional areas for the entire set of species within Europe was variable. Across the 4 scenarios of climate change analyzed, species averaged 2.4–3.1% decline in potential distributional area (Table 2). The Hadley and Canadian scenarios yielded projections that were closely similar, with the Canadian datasets slightly less drastic in projections than the Hadley datasets (Figure 2). The “A” scenarios, however, were considerably more drastic in their projections than the “B” scenarios. Interestingly, the former scenarios predicted both more drastic expansions and more drastic contractions, as is noticeable in the broader variation in predicted area losses and gains (Figure 2).

**Table 2:** Summary of projected climate change effects on native ranges of European plants, as well as on modeled potential invasive ranges on other continents, as a function of 4 different models and scenarios of future (2055) climates.

	Europe	Africa	Asia	Australia	North America	South America
Canadian CGCM1a2	−2.445 (7.063)	−4.554 (21.236)	−0.377 (2.085)	−0.352 (8.272)	−0.365 (1.532)	0.106 (4.372)
Canadian CGCM1b2	−2.676 (4.933)	−3.060 (18.403)	−0.534 (1.610)	−0.147 (6.752)	−0.499 (1.119)	−0.163 (4.141)
Hadley HadCM3a2	−3.096 (6.903)	−5.737 (24.329)	−0.676 (1.643)	−0.335 (8.604)	−0.420 (1.126)	0.023 (4.315)
Hadley HadCM3b2	−2.611 (5.897)	−4.361 (21.549)	−0.627 (1.438)	−0.105 (6.761)	−0.250 (0.993)	0.053 (4.202)
Species projected to increase in potential distributional area	651	1324	435	1504	406	1438

Numbers presented are average percent change relative to projected present potential distributional areas (standard deviations in parentheses). The final row presents numbers of species projected to increase in potential distributional area based on an average of all 4 future-climate scenarios, out of a possible 1804 species.

Looking at projections of change in potential distributional areas for each species as an invasive on continents beside Europe, Southern Hemisphere continents tended to have most species increasing in invasive potential, whereas Northern Hemisphere continents generally saw species decreasing somewhat in invasive potential (Table 2). Africa showed greatest losses in potential area (Figure 2; Table 2), but South America shows a more mixed response—increasing in potential distributional area under 3 scenarios and decreasing under one (Canadian B scenario). Curiously, all three Southern Hemisphere continents have average declines in potential area, yet around two-thirds of species increased in habitable area, suggesting that the declining third declined dramatically (Table 2).

**Figure 3:**
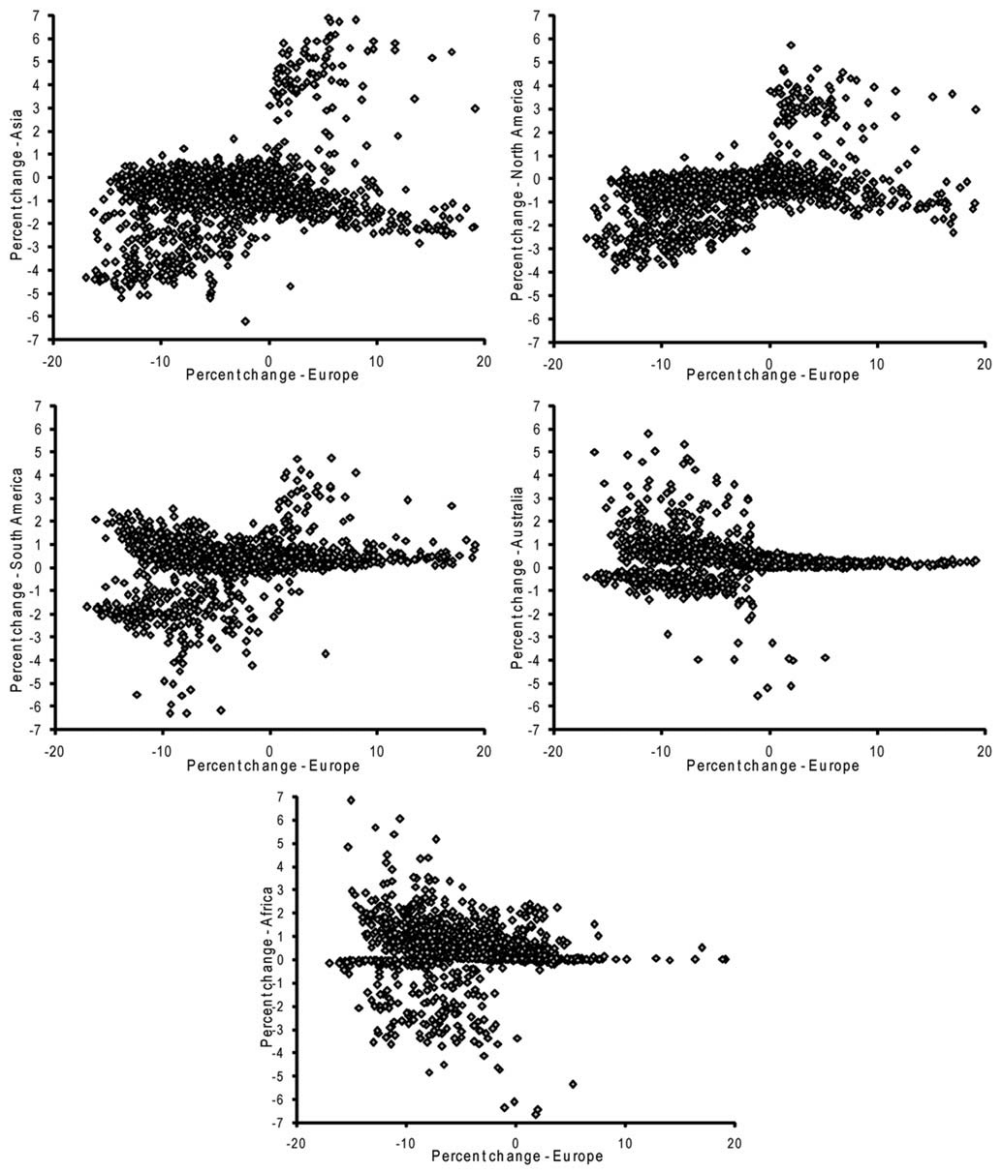
Summary of relationship between projected changes in distributional area on the native (European) distributional area and projected changes in potential distributional area in nonnative areas on five other continents.

Changes in potential distributional area in Europe (native range) relate to potential distributional area on other continents in odd ways (Figure 3). In general, species increasing in potential distributional area in Europe also increased in distributional potential on Northern Hemisphere continents, but relationships were more complex in Southern Hemisphere continents—species declining in potential distributional area in Europe might increase or decrease in potential distributional area in the Southern Hemisphere, but species increasing in European potential distributional area generally showed no change in potential distributional area in the Southern Hemisphere.

## Discussion

In general, most European plant species are anticipated to decline somewhat in their invasive potential on other continents. Because many European plant species reach their tolerance limits for warm climates in southern Europe, further warming of climates is unlikely to allow these species to invade broadly into the Subtropics and Tropics, and rather are pushed farther north. With northward shifts, most species see shrinking distributional areas. As a consequence, species with shrinking distributional potential in Europe should also see reductions in potential distribution in North America and Asia as well, whereas translation of these effects into the Southern Hemisphere may prove more complex. Similarly, a recent study of Argentine ant global invasive potential [11] found global invasive potential declining somewhat, but with potential for some regional expansions. Nonetheless, more European species were predicted to see expanded possibilities in the Southern Hemisphere, but those declining declined dramatically, thus creating opposing signals.

If nothing else, this study serves to illustrate the complexities of the likely effects of climate change on biodiversity. Even the relatively simple native-range projections that have been made about climate change effects on biodiversity have been complex—landscapes, taxa, and particular climate change scenarios all affect the predicted implications of climate change for biodiversity [13], [20], [36], [37], [38], [39]. The early, crude generalities that were made about how climate change will affect species’ invasions [10] clearly underestimated this complexity.

This study does—of course—have limitations. Most prominently, the 50×50 km resolution of the European occurrence data on which the models were based limits results to a fairly generalized characterization of niches of species. Second, Europe being a fairly small region and one without great environmental diversity, these analyses may not illustrate the full diversity of likely responses by species to climate change processes. Finally, focusing only on plants may also limit the diversity of phenomena that can be appreciated in a survey such as this one.

Still, the general picture painted herein is probably robust. That is, the invasive potential of species in the face of changing climates is very complex, and is not immediately predictable based on simple generalizations. Individual species each have their own particular ecological needs, and those needs make for distinct patterns of invasive potential. This individuality parallels that observed in studies of climate change effects on native species [37], and is echoed even more clearly in our explorations of interactions between climate change effects and invasive potential of species. In this broad survey, invasive potential on average declined with warming climates, yet particular species in certain nonnative regions actually increased in potential distributional area.

## Methods

### Input Occurrence Data

Distributional data were available for 2362 plant species [40], [41], comprising ∼20% of the total European flora, sampled between 1972 and 1996. Although not all of the species in the European flora compilation are actually native to Europe, the great majority was, and as such we treat the European continent as a source area for invasive plant populations. The data have a bias towards well-represented groups in western and central Europe whereas taxa in the Mediterranean region are relatively underrepresented [42]. These data document species distributions on a UTM (Universal Transverse Mercator) 50×50 km grid covering most of Europe, but excluding Russia and the Caucasus, where survey effort was less intensive. This data set has seen numerous detailed analyses, including several based on ENM [20], [35]. Of the 2362 plant species in the overall data set, we developed predictive models for all 1804 species for which ≥20 occurrences (i.e., grid squares) were available.

### Environmental Data Sets

To characterize present-day climates, we used coarse-scale climate summaries [43], including annual mean precipitation, annual mean temperature, annual mean maximum monthly temperature, and annual mean minimum monthly temperature (more dimensions are available, but this reduced set was necessary owing to the more limited suite of dimensions available for future climates). Future climates were summarized via parallel data sets summarizing general circulation model results (projection to 2055) drawn from the Hadley (HadCM3) and Canadian (CGCM1) climate modeling centers [44], [45], in each case for the A2 and B2 emissions scenarios—these two scenarios bracket the range of most likely future climate conditions (i.e., being relatively liberal and relatively conservative, respectively). Future climate data sets were obtained from the Data Distribution Centre of the Intergovernmental Panel on Climate Change [46]. As such, we analyzed species’ global potential distributions under present-day conditions and 4 scenarios of future conditions (HadCM3 A2 and B2, CGCM1 A2 and B2).

### Ecological Niche Modeling

Several studies have compared projections by different models, concluding that complex-fitting algorithms provide generally better projections than simpler analogues [47], [48]. In these studies model performance was assessed using measures of model fit, i.e., measuring how well models fitted the training data or test data on the same landscape as the training data. The problem addressed in this contribution is that of transferability – making projections into situations that are statistically independent from the training data, and such measures of model performance have been shown to be overly optimistic [38]. In practice and despite all model comparisons performed in recent years, little guidance can be provided regarding the selection of ‘best’ ENM algorithms for transferability [49]. Here, we used the Genetic Algorithm for Rule-Set Projection (GARP), a method that has been extensively used in studies involving transferability [26], [49]. The OpenModeller version of the GARP [50], [51] was utilized. GARP is an evolutionary-computing method that builds ENMs based on nonrandom associations between known occurrence points for species and sets of GIS coverages describing variation in several ecological parameters of environments. Occurrence data are used by GARP as follows: 50% of occurrence data points are set aside for an independent filtering to assure predictive ability of models (extrinsic testing data), 25% are used for developing models (training data), and 25% are used for tests of model quality internal to GARP (intrinsic testing data). Distributional data are converted to binary raster layers, and by random resampling from training and intrinsic test data and areas of ‘pseudoabsence’ (areas lacking known presences), two data sets are created, each of 1250 points; these data sets are used for rule generation and model testing, respectively [50], [51].

Within GARP’s processing, the first rule is created by applying a method chosen randomly from a set of inferential tools (e.g., logistic regression, bioclimatic rules). The genetic algorithm consists of specially defined operators (e.g. crossover, mutation) that modify the initial rules, and thus the result are models that have “evolved”—after each modification, the quality of the rule is tested (to maximize both significance and predictive accuracy) and a size-limited set of best rules is retained. Because rules are tested based on independent data (the intrinsic test data), performance values reflect expected performance of rules, an independent verification that gives a more reliable estimate of true rule performance.

The result is a set of rules that can be projected onto a map to produce a potential geographic distribution for the species under investigation—in this particular study, we projected models trained within Europe based on European occurrences and European environmental data to worldwide coverages for present-day and 4 future-climate coverage sets. For all analyses, we used the OpenModeller interface to the GARP algorithm that has been optimized for use in parallel- and grid-computing environments [52], and carried out the modeling on a 128-processor high-throughput parallel computing cluster at the University of Kansas. All model output was in the form of geo-tif image files.

Following recent best-practices recommendations [53], for each species, we developed 100 replicate random-walk GARP models, and filtered out 90% based on consideration of error statistics, as follows. The ‘best subsets’ methodology consists of an initial filter removing models that omit (omission error  =  predicting absence at points of known presence) heavily based on the extrinsic testing data, and a second filter based on an index of commission error ( =  predicting presence in areas of known absence), in which models predicting very large and very small areas are removed from consideration. Specifically, in GARP, we retained only the 20% of models that showed lowest omission errors, and then retained only the central 50% of the frequency distribution of proportional area predicted present (an index of commission error); the result was 10 ‘best subsets’ models (binary raster data layers) that were summed to produce a best ensemble estimate of geographic projection.

### Evaluating Model Projections

Model projections were tested for 10 European plant species already invasive in North America (see Text S1) to assess the predictive ability of these models, based on the example of a previous publication [29], as follows. In these tests, the data used to validate models are completely independent of the data used to train them, as they are exclusively on another continent from where the model was trained [38]. We drew North American occurrence data from the U.S. Department of Agriculture’s *Plants Database* [54], summarizing known ranges of each species at the level of counties in which they are known to occur by means of adding fields for each species to the attributes table of a vector dataset summarizing boundaries of 3111 counties across the United States.

To summarize projections from the ecological niche model for each species in each county, we plotted 5 random points within each county, and intersected those points with the raster grid from the ENM; we tallied a county as ‘predicted present’ if ≥1 of its random points intersected areas of predicted presence. We calculated (1) the number of counties where the species is known to occur (*x*), (2) the number of counties in which the species was predicted to occur (*y*), and the number of counties in which the known occurrence coincides with a county of predicted occurrence (*z*). From these 3 quantities, we calculated the cumulative binomial probability of obtaining *z* successes, given *x* trials, and with a *y*/3111 probability of success. This calculation, in effect, provides a one-tailed probability of achieving the observed level of coincidence between known and predicted invasive distributional areas by chance alone.

### Analysis and Interpretation

Worldwide projections for present and 4 future climate scenarios were summarized as follows. To summarize patterns on more specific spatial scales, we subset the global output grids to focus on each continent except Antarctica (Europe, Africa, Asia, Australia, North America, South America), and calculated areas predicted present in each by reprojecting GARP results from geographic coordinates to a Lambert Equal Area projection, and extracting areas predicted present. Final area estimates were in terms of m^2^ predicted present on each continent under each climate scenario, which were translated into calculations of percent change between future and present-day climates.

## Supporting Information

Text S1 European Plant Species in Occurrence Data Set and Invasive in North America
(0.02 MB DOC)
