# Supplementary material for: Shifting Global Invasive Potential of European Plants with Climate Change
Source: PLoS One. 2008 Jun 18;3(6):e2441. doi: 10.1371/journal.pone.0002441 (PMC2409072; doi:10.1371/journal.pone.0002441)
Supplement: Text S1 — European Plant Species in Occurrence Data Set and Invasive in North America (0.02 MB DOC) [file pone.0002441.s001.doc]

*Alyssum alyssoides* (L.) L., *Arabidopsis thaliana*, *Barbarea vulgaris* Ait. f., *Berteroa incana* (L.) DC., *Brassica rapa* L., *Brassica tournefortii* Gouan, *Capsella bursa-*pastoris (L.) Medik., *Cardamine hirsuta* L., *Cardamine impatiens* L., *Cardamine parviflora* L., *Coronopus didymus* (L.) Sm., *Coronopus didymus* (L.) Sm., *Coronopus squamatus* (Forssk.) Aschers., *Descurainia sophia* (L.) Webb ex Prantl, *Erysimum cheiranthoides* L, *Hesperis matronalis* L., *Isatis tinctoria* L., *Lepidium campestre* (L.) Ait. f., *Lepidium latifolium* L., *Rorippa austriaca* (Crantz) Bess., *Rorippa sylvestris* (L.) Bess., *Sinapis alba* L., *Sisymbrium altissimum* L., *Sisymbrium irio*, *Sisymbrium loeselii* L., *Thlaspi arvense* L., *Amaranthus blitoides* S. Wats, *Amaranthus spinosus* L., *Amaranthus viridus* L., *Carpobrotus edulis* (L.) L. Bolus, *Cerastium fontanum*, *Chenopodium album* L, *Chenopodium ambrosioides* L., *Chenopodium murale* L., *Claytonia perfoliata* ssp. *perfoliata*, *Clematis orientalis* L., *Clematis vitalba* L., *Dianthus armeria* L, *Lychnis flos-cuculi* L., *Mollugo verticillata* L., *Sagina procumbens* L., *Salsola kali* L., *Salsola vermiculata* L., *Saponaria officinalis* L., *Scleranthus annuus* L., *Silene conoidea* L., *Silene latifolia* Poir, *Silene noctiflora* L., *Silene vulgaris* (Moench) Garcke, *Spergula arvensis* L., *Stellaria graminea* L., *Stellaria media* (L.) Vill., *Papaver dubium* L., *Papaver somniferum* L., *Polygonum aviculare* L., *Polygonum persicaria* L., *Rumex maritimus* L., *Rumex obtusifolius* L., *Berberis vulgaris* L., *Ranunculus acris* L., *Ranunculus bulbosus* L., *Ranunculus ficaria* L., *Ranunculus repens* L., *Ranunculus sardous* Crantz.
